# The Epidemiology and Outcomes of Septic Arthritis in the Maltese Islands: A Hospital-Based Retrospective Cohort Study

**DOI:** 10.31138/mjr.31.2.195

**Published:** 2020-06-30

**Authors:** Christian Vassallo, Andrew A. Borg, Daniel Farrugia, Cecilia Mercieca

**Affiliations:** 1 Department of Rheumatology; 2 Department of Pathology, Mater Dei Hospital, Msida, Malta

**Keywords:** Epidemiology, infection, septic arthritis, mortality, risk factors

## Abstract

**Objective/Aim:**

Septic arthritis is an uncommon but important disease with significant morbidity and mortality, especially if inadequately managed. The aim of this epidemiological study was to identify the characteristics and outcomes of patients treated for septic arthritis at Mater Dei Hospital, Malta, over a 10-year period.

**Methods:**

This was a retrospective observational study. Patients diagnosed with septic arthritis between 2008 and 2018 were recruited. Cases were identified by reviewing all inhospital episodes of patients diagnosed with septic arthritis according to Newman criteria.

**Results:**

There were 124 cases of native joint septic arthritis and 138 of prosthetic joint infection. Cases were present amongst all age groups, with the highest incidence amongst those aged 61–70 years for both native and prosthetic infections. Fever was present in around 40% of cases. Raised white cell count was prevalent in 66.9% of native joint infections and 52.9% of prosthetic joints. Elevated C-reactive protein was overwhelmingly seen in most cases, present in 93.5% (median=159.5 mg/L; IQR=85.8–291) of native joints and 92.0% of prosthetic joint infections (median=68.7 mg/L; IQR=20.5–186). Over 55% of patients had one or more risk factors for joint sepsis, diabetes mellitus being the most prevalent clinical comorbidity (22.6% and 24.6% for native and prosthetic joint infections respectively). Synovial cultures were positive in 66% and 82% of native and prosthetic joint aspirates respectively. *Staphylococcus aureus* was the most commonly isolated organism from both native and prosthetic joint infection, followed by streptococcal infections in native joints and coagulase negative staphylococci and gram-negative infections in prosthetic joints. Fifteen deaths were directly attributed to joint sepsis.

**Conclusion:**

Absence of fever and elevated white cell count does not exclude the diagnosis. The mortality rate due to septic arthritis in this cohort of patients was found to be 5.7%. All deaths occurred in elderly patients with clinical comorbidities suggesting that this group is at highest risk.

## INTRODUCTION

Septic arthritis is the most serious cause of an inflamed, swollen joint.^1–4^ It has significant morbidity and mortality. Incidence varies between 2–10 per 100,000 patient years in the USA and Western Europe.^4–6^ The diagnosis is challenging and often delayed even for doctors experienced in the management of musculoskeletal medicine. Timely diagnosis and targeted treatment are imperative as inadequately treated infections may result in irreversible joint destruction with consequent long-term disability.^7,8^ Although joint aspiration and positive cultures are useful to pin the diagnosis, septic arthritis often occurs in the setting of negative cultures, particularly in patients already taking antibiotics prior to aspiration or in the case of fastidious organisms. Culture negative septic arthritis poses a particular challenge as there is no diagnostic test with sufficient sensitivity or specificity to reliably distinguish between bacterial and non-bacterial sources for inflammation.^9,10^

There is sparse epidemiological and outcome data on septic arthritis in different populations as the rarity and nature of the disorder often means that prospective studies are technically difficult to carry out. Such information could help with identifying clinical patterns of disease and management.

## MATERIALS AND METHODS

This was a retrospective observational study. Medical case records for all patients admitted with a diagnosis of septic arthritis to Mater Dei Hospital, Malta from August 2008 to December 2018 were analysed. No patients were enrolled into this study. Mater Dei Hospital is a teaching hospital affiliated with the University of Malta; it is an acute tertiary centre which caters for the entirety of the Maltese population, with dedicated microbiology and infectious disease teams for clinical guidance. Cases treated for septic arthritis were identified by analysing all in-patient medical records with the following ICD-10-CM codes:

- M00 Pyogenic arthritis;
- M01 Direct infections of joint in infectious and parasitic diseases classified elsewhere;
- T84.5 Infection and inflammatory reaction due to internal joint prosthesis;
- T84.6 Infection and inflammatory reaction due to internal fixation device [any site];
- T84.7 Infection and inflammatory reaction due to other internal orthopaedic prosthetic devices, implants and grafts.

Data was obtained in accordance to local data protection policies with the aid of the Malta Health Information and Research Department. All medical records were processed without disclosing patients’ identities. Patients’ case notes were individually reviewed in a systematic manner with details recorded on a standardized electronic form.

A total of 429 cases were traced over the 10-year period. Patients were classified according to the Newman criteria^11^; cases were considered as confirmed if they had an organism isolated from infected synovial fluid (Grade A), organisms isolated from elsewhere (Grade B) or no organism isolated but with clinical examination and investigations strongly suggestive of septic arthritis (Grade C). Patients included must have undergone full treatment for joint sepsis. Furthermore, those patients classified as grade C required an elevated white cell count (WCC) and/or fever and/or the presence of an acute phase response (raised erythrocyte sedimentation rate (ESR) and/or C-reactive protein (CRP)), in addition to an acutely inflamed and swollen joint. Cases of osteomyelitis without joint involvement were excluded.

167 cases were subsequently excluded from this study following case review as these either had alternative diagnoses or did not meet the inclusion criteria. Data extracted included the following: demographics, clinical presentation, concomitant conditions and risk factors for joint sepsis, investigations, microbiology data, antibiotic regimens, surgical procedures and outcomes. Cases of native and prosthetic joint infections (PJIs) are reported separately. Results are summarized in *Tables 1–4* for ease of reference.

**Table 1.** Demographics and clinical characteristics for native and prosthetic joints.

***Patient Demographics***			
	**Native Joint**	**Prosthetic Joint**	**p-value**
***Patient Demographics***			
Number of cases	124	138	-
Median (IQR) age in years	66 (41.3–75)	69 (63–77)	<0.001
Percentage of males	62.1%	48.6%	0.034
Number (%) of deaths	12 (9.7%)	13 (9.4%)	1.000
Number (%) of deaths attributed to joint sepsis	7 (5.6%)	8 (5.8%)	1.000
***Number of patients according to Newman Criteria (n= (%))***			
Newman Grade A	82 (66.1%)	113 (81.9%)	0.004
Newman Grade B	6 (4.8%)	8 (5.8%)	0.789
Newman Grade C	36 (29.0%)	17 (12.3%)	0.001
***Joints affected (n= (%))***			
Knee	84 (67.7%)	86 (62.3%)	0.368
Shoulder	12 (9.7%)	2 (1.4%)	0.004
Hip	7 (5.6%)	50 (36.2%)	<0.001
Elbow	5 (4.0%)	0	0.023
Others	16 (12.9%)	0	<0.001
***Presentation (n= (%))***			
Mean (±SD) time to presentation	8.3 (±6.5) days	6.67 (±6) days	0.021
Median (IQR) time to presentation	6 (2–14) days	4.5 (2–14)	0.021
History of acutely hot and swollen joint	111 (89.5%)	112 (81.2%)	0.081
Fever	50 (40.3%)	55 (39.9%)	1.000
Limitation in joint movement	102 (82.3%)	105 (76.1%)	0.229
Leucocytosis (WCC >10×10^9^/L )	83 (66.9%)	73 (52.9%)	0.024
Mean (±SD) WCC (×10^9^/L )	11.8 (±3.1)	10.9 (±4.6)	0.001
Median (IQR) WCC (×10^9^/L )	12.2 (9.7–13.5)	10.2 (7.6–12.8)	0.001
Raised inflammatory markers	118 (95.2%)	134 (97.1%)	0.524
Raised CRP (>5mg/L)	116 (93.5%)	127 (92.0%)	0.812
Median (IQR) CRP (mg/L)	159.5 (85.8–291)	68.7 (20.5–186)	<0.001
Raised ESR (>30mm 1^st^ hr )	104 (83.9%)	116 (84.1%)	1.000
Mean (±SD) ESR (mm 1^st^ hr )	74.6 (±37.9)	73.6 (±36.6)	0.779
Median (IQR) ESR (mm 1^st^ hr )	70 (39–113)	83 (39–108)	0.779
***Clinical co-morbidities and precipitating factors (n= (%))***			
Osteoarthritis affecting infected joint	38 (30.6%)	-	-
Diabetes mellitus	28 (22.6%)	34 (24.6%)	0.771
Cutaneous ulcers	10 (8.1%)	13 (9.4%)	0.828
Direct trauma prior to presentation	17 (13.7%)	12 (8.7%)	0.238
Concomitant oral glucocorticoids	8 (6.5%)	6 (4.3%)	0.584
Rheumatoid arthritis (RA)	2 (1.6%)	8 (5.8%)	0.108
Psoriasis	2 (1.6%)	0	0.223
Illicit intravenous drug use	7 (5.6%)	0	0.005
Recent intra-articular glucocorticoid injection	3 (2.4%)	0	0.105
Recent hospitalization	37 (29.8%)	90 (65.2%)	<0.001

CRP, C-Reactive protein; ESR, Erythrocyte sedimentation rate; WCC, White cell count.

**Table 2.** Summary of organisms cultured. Percentages for each organism cultured are based on total numbers of positive cultures for each group.

	**Native Joint**	**Prosthetic Joint**	**p-value**
***Isolated Organisms (n= (%))***			
Total number of positive cultures (Grades A+B)	88 (71.0%)	121 (87.7%)	0.001
Staphylococcus aureus	54 (61.4%)	68 (56.2%)	0.480
*MSSA*	32 (36.4%)	43 (35.5%)	1.000
*MRSA*	22 (25.0%)	25 (20.7%)	0.504
Streptococcus spp.	15 (17.0%)	15 (12.4%)	0.425
Gram negatives	14 (15.9%)	28 (23.1%)	0.224
*Pseudomonas spp.*	5 (5.7%)	10 (8.3%)	0.592
*Escherichia coli*	4 (4.5%)	9 (7.4%)	0.564
*Klebsiella spp.*	3 (3.4%)	10 (8.3%)	0.245
*Roseomonas mucosa*	1 (1.1%)	0	0.421
*Proteus mirabilis*	0	4 (3.3%)	0.140
Coagulase negative Staphylococci	4 (4.5%)	28 (23.1%)	<0.001
Other gram positives	5 (5.7%)	8 (6.6%)	1.000
Mixed infection	7 (8.0%)	18 (14.9%)	0.138

MSSA, Methicillin sensitive Staphylococcus aureus; MRSA, Methicillin resistant Staphylococcus aureus.

**Table 3.** Summary of treatments used.

	**Native**	**Prosthetic**	**p-value**
***Duration of antibiotics***			
Median (IQR) duration of parenteral antibiotics	14 (10–21) days	14 (14–22) days	0.103
Median (IQR) duration of oral antibiotics	14 (14–28) days	90 (42–180) days	<0.001
***Antimicrobials Used (n= (%))***			
Ciprofloxacin	48 (38.7%)	44 (31.9%)	0.300
Clindamycin	31 (25.0%)	25 (18.1%)	0.179
Co-amoxiclav	19 (15.3%)	15 (10.9%)	0.358
Flucloxacillin	30 (24.2%)	12 (8.7%)	<0.001
Levofloxacin	15 (12.1%)	53 (38.4%)	<0.001
Rifampicin	10 (8.1%)	74 (53.6%)	<0.001
Teicoplanin	18 (14.5%)	45 (32.6%)	<0.001
***Interventions Performed (n= (%))***			
Closed needle aspiration	82 (66.1%)	-	-
Joint drainage and washout	60 (48.4%)	-	-
Synovectomy	14 (11.3%)	-	-
DAIR	-	73 (52.9%)	-
Revision surgery	-	42 (30.4%)	-
Excision arthroplasty	-	11 (8.0%)	-

DAIR, Debridement, antibiotics and implant retention.

**Table 4.** Characteristics of patients who died from joint sepsis.

***Patient***	***Age***	***Gender***	***Medical history***	***Affected Joint***	***Bacterium***
1	86	F	DM; CHF	Finger	*Staphylococcus aureus*
2	86	M	DM; IHD; Hypertension	Shoulder	*Staphylococcus aureus*
3	87	F	DM; CHF; Hypertension	Knee	*Staphylococcus aureus*
4	80	F	DM; Peripheral vascular disease; Hypertension	Shoulder	*Streptococcus spp.*
5	81	M	Hypertension	Knee prosthesis	*Streptococcus spp.*
6	68	F	DM; CHF; Hypertension; End Stage Kidney Disease on haemodialysis	Knee	*Staphylococcus aureus*
7	81	F	DM; Rheumatoid arthritis; Hypertension	Hip prosthesis	*Staphylococcus aureus*
8	67	M	IHD; Hypertension	Knee prosthesis	*Staphylococcus aureus*
9	85	M	DM	Shoulder	*Staphylococcus aureus*
10	75	M	DM; CHF; IHD; Hypertension; CKD	Knee prosthesis	*Staphylococcus aureus*
11	67	M	DM; CHF; CKD	Knee prosthesis	*Staphylococcus aureus*
12	87	M	Hypertension	Knee prosthesis	*Staphylococcus aureus*
13	86	F	DM; Osteoporosis; Dementia	Knee prosthesis	*Escherichia coli; Streptococcus spp.; Pseudomonas aeruginosa; Bacteroides fragilis*
14	85	M	DM; Hypertension; CKD; CHF; IHD	Knee	*MRSA*
15	80	M	Hypertension; Paget’s disease; dementia	Hip prosthesis	*Staphylococcus aureus*

CHF, Congestive heart failure; CKD, Chronic kidney disease; DM, Diabetes mellitus; IHD, Ischaemic heart disease.

Normality testing was carried out using the Shapiro-Wilk test. Measures of central tendency are presented as means (with standard deviations) or medians (with interquartile ranges), depending on normality of distribution. Nominal variables are shown as frequencies with percentages. Categorical variables were compared using Fisher’s exact test and metric variables were compared using the Mann-Whitney U test. A two-sided p-value of <0.05 was considered statistically significant. Data was analysed using the Statistical Program for Social Science (SPSS) V.26 (IBM).

## RESULTS

### Patients with native joint infections

**Figure 1.**
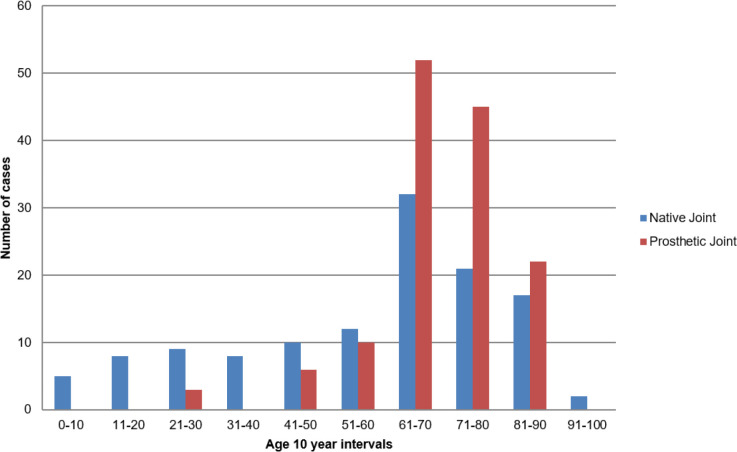
Age distribution of septic arthritis cases.

There were 124 cases of native joint infection, 77 were males and 47 were females. The median age was 66 (IQR=41.3–75) years. In terms of the Newman criteria, 82 patients (66.1%) were classified as grade A, 6 patients (4.8%) as grade B and 36 (29.0%) as grade C. Cases of native joint infection were identified across all age groups with the highest proportion (32 cases, 25.8%) in the 61–70 year age group (*Figure 1*). 22 (17.7%) of these patients had already been given antibiotics prior to obtaining synovial and blood cultures, while in 7 cases it was unclear whether antibiotics were administered before cultures were taken.

84 patients presented with knee joint infections, followed by 12 with infected shoulders, 7 infected hip joints, 5 infected elbow joints and 16 with other joint infections. Only one polyarticular case was identified. Six (4.8%) male paediatric cases were identified (range= 4–15 years), 3 cases involved sepsis of the hip joint. One female paediatric case of septic knee arthritis was identified. In terms of presentation, a history of an acutely hot and swollen joint was reported in 111 cases (89.5%), fever (>36.7°C) in 50 (40.3%) and limitation of joint movement in 102 patients (82.3%). The median time to presentation was 6 (IQR=2–14) days. Leucocytosis (total WCC >10.0×10^9^/L) was reported in 83 (66.9%) patients with a mean (±SD) WCC of 11.8 ±3.1 ×10^9^/L. Raised inflammatory markers were seen at presentation in 118 (95.2%) cases; 116 (93.5%) patients had elevated CRP (>5mg/L) (median 159.5 (IQR= 85.8–291); and 104 (83.9%) patients had raised ESR (>30mm 1^st^ hr) (mean (±SD) 74.6 ± 37.9mm 1^st^ hr).

One or more clinical co-morbidities were observed in 80 patients (64.5%). The commonest comorbidity was osteoarthritis affecting the infected native joint (38 cases, 30.6%), followed by diabetes mellitus (28 cases, 22.6%). Other relevant clinical comorbidities and precipitating factors present in this cohort of patients are summarized in *Table 1*.

The most common isolated organism was *Staphylococcus aureus*, cultured from 54 joint aspirates (61.4%). 22 of these (25.0%) of these showed resistance to methicillin (MRSA). Other gram positive and negative organisms cultured are displayed in *Table 2*. No cases of gonococcal infection were reported in this study. A case of tuberculous septic arthritis was diagnosed in a 26-year-old African immigrant who had recently arrived in Malta; this was his first presentation of tuberculosis and had reported a 30-day history of knee pain and swelling. The median duration of parenteral antimicrobial treatment for native joint septic arthritis was 14 (IQR=10–21) days followed by 14 (IQR=14–28) days for oral antibiotics. Antimicrobials were used in different combinations - ciprofloxacin was the most common antibiotic used (48 cases, 38.7%), followed by clindamycin (31 cases, 25.0%), flucloxacillin (30 cases, 24.2%) and co-amoxiclav (19 cases, 15.3%). Closed needle aspiration was performed in 82 cases (66.1%); 60 infected joints (48.4%) were drained and washed out either arthroscopically or via open surgery and 14 synovectomies (11.3%) were performed. Treatments administered are summarized in *Table 3*.

12 deaths (9.7%) were reported to have occurred, of these 7 (5.6%) were directly attributed to joint sepsis. Concomitant conditions and bacteria isolated in these patients are highlighted in *Table 4*.

Other causes for death included one case each of bronchopneumonia, bowel ischaemia, cerebrovascular accident, lung carcinoma and upper gastrointestinal bleed.

### Patients with prosthetic joint infections (PJIs)

138 cases of PJI were identified, 67 of which were male and 71 were female. The median age was 69 (IQR=63–77) years. According to the Newman criteria, 113 patients (81.9%) were classified as grade A, 8 patients (5.8%) as grade B and 17 (12.3%) as grade C. The highest incidence for PJI was also found to be in the 61–70-year age group (*Figure 1*).

86 patients presented with prosthetic knee joint infection, 50 cases involved prosthetic hip infections and 2 patients presented with prosthetic shoulder infections. A history of a hot, swollen joint was reported in 112 cases (81.2%), fever (>36.7°C) in 55 (39.9%) and limited joint movement in 105 patients (76.1%). The mean (±SD) time to presentation was 6.67 (±6) days. Leucocytosis was observed (total WCC >10.0×10^9^/L) in 73 (52.9%) tested patients with a median WCC of 10.2 (IQR=7.6–12.8) ×10^9^/L. Markers of inflammation were raised in 97.1% of patients, with 127 (92.0%) patients having raised CRP (>5mg/L) (median 68.7 (IQR=20.5–186) mg/L) and 116 (84.1%) patients having an elevated ESR (>30mm 1^st^ hr) (median 83 (IQR=39–108) mg/L).

The presence of at least one clinical co-morbidity was observed in 78 patients (56.5%), the commonest being diabetes mellitus (34 cases, 24.6%). In this cohort, no patients had a history of illicit intravenous drug use. The rates of other clinical co-morbidities and precipitating factors are displayed in *Table 1*.

*Staphylococcus aureus* was again the commonest isolated organism (68 cases, 56.2%), over a third of which were MRSA (25 cases, 20.7%). 28 cultures (23.1%) were positive for coagulase negative Staphylococci (CoNS) and another 28 for gram negative organisms, the majority of which were Enterobacteriaceae. Other isolated gram positive and negative organisms are summarized in *Table 2*.

The median duration of antimicrobial treatment for PJI was 14 (IQR=14–22) days for intravenous antibiotics followed by 90 (IQR=42–180) days for oral treatment. Various antimicrobials in different combinations were used, with rifampicin (74 cases, 53.6%) and levofloxacin (53 cases, 38.4%) being the commonest two, followed by teicoplanin (45 cases*,* 32.6%) and ciprofloxacin (44 cases, 31.9%). The most common initial antibiotic combination was teicoplanin and a quinolone (38 cases, 27.5%). DAIR (Debridement, antibiotics and implant retention) was the commonest procedure performed in 73 patients (52.9%) followed by revision surgery (42 cases, 30.4%) and excision arthroplasty (11 cases, 8.0%). A summary of treatments used is provided in *Table 3*.

13 deaths (9.4%) occurred in patients being treated for PJIs, 8 (5.8%) of these were directly attributed to joint sepsis (*Table 4*). Other causes for death encountered in our cohort of PJI patients included two patients with bronchopneumonia and one each with metastatic renal cell carcinoma, congestive heart failure and cerebrovascular accident.

### Patients with native joint infections vs PJIs

When directly compared, a significantly higher proportion of males were seen in our native joint cohort (62.1% vs 48.6% for PJIs; p= 0.034). Statistically significant differences were also seen between the ages for both groups (p= <0.001), despite similar median values (66 years for native infections vs 69 years for PJIs), due to the higher incidences of native joint sepsis occurring in younger individuals (*Figure 1*). Mortality attributed to joint sepsis (5.6% for native infections vs 5.8% for PJIs; p= 1.000) was similar between both patient groups.

Significantly higher rates for Newman grade A infections were treated in the PJI group versus the native group (81.9% vs 66.1% respectively; p= 0.004); the inverse was encountered for Newman grade C patients, which were significantly higher in native joint sepsis (29.0% vs 12.3% for PJIs; p= 0.001).

Similar proportions of knee infections were encountered for both cohorts (67.7% for native vs 62.3% for PJIs; p= 0.368), with much lower percentages seen with native hip infections vs prosthetic hips (5.6% vs 36.2% respectively; p= <0.001). Both patient cohorts had very similar overall presentations and biochemical findings; discernible differences encountered included the higher percentage of leucocytosis seen with native joint sepsis (66.9% vs 52.9% in PJI group; p= 0.024) together with higher median WCC and CRP values when compared to the PJI cohort; (WCC 12.2×10^9^/L vs 10.2×10^9^/L respectively; p= 0.001; CRP 159.5mg/L vs 68.7mg/L respectively; p= <0.001).

Clinical co-morbidities and precipitating factors were very similar between native and prosthetic infections across the board, with no statistical differences encountered for the presence of diabetes mellitus (22.6% vs 24.6% respectively; p= 0.771), cutaneous ulcers (8.1% vs 9.4% respectively; p= 0.828), direct trauma (13.7% vs 8.7% respectively; p= 0.238), concomitant glucocorticoids (6.5% vs 4.3% respectively; p= 0.584) or RA (1.6% vs 5.8% respectively; p= 0.108). Significant differences were encountered in terms of patients with a history of illicit drug use in the native joint vs PJI group (5.6% vs 0% respectively; p= 0.005). Significantly higher proportions of patients with PJIs were also seen to have been recently hospitalized when compared to native joint patients (65.2% vs 29.8% respectively; p= <0.001).

Staphylococcus aureus was the most commonly encountered organism in both groups, with no significant difference in their proportions (61.4% for native infections vs 56.2% for PJIs; p= 0.480). Apart from infections secondary to CoNS (4.5% for native infections vs 23.1% for PJIs; p= 0.0002), no other significant differences were seen for isolated organisms between the two cohorts.

In terms of treatments used, significantly higher proportions of PJI patients were treated with levofloxacin (38.4% vs 12.1% for native infections; p= <0.001), rifampicin (53.6% vs 8.1% for native infections; p= <0.001) and teicoplanin (32.6% vs 14.5% for native infections; p= <0.001), whilst a higher proportion of native joint infections were treated with flucloxacillin (24.2% vs 8.7% for PJIs; p= <0.001). Differences in terms of approaches to surgical management for both patient groups meant that a direct comparison between the two was not possible.

## DISCUSSION

Septic arthritis is an uncommon but important medical emergency with significant morbidity and mortality, especially in the elderly.^9^ To our knowledge this is the first epidemiological study of septic arthritis carried out in the Maltese Islands. Septic arthritis was traditionally described to be a disease that arises mainly in the elderly and very young children.^1–3^ This study exhibited a higher incidence in patients over the age of 60 and only 7 paediatric cases (<16 years of age) were seen during the ten year period: this adds support to more recent literature which demonstrates that joint sepsis is no longer a disease with great predominance in the paediatric population.^12–14^

The typical symptoms at presentation of septic arthritis include an acutely hot, swollen joint with reduced range of movement. Affected individuals are often febrile at presentation, although chills and spiking fevers are not always present.^15^ The presence of fever on presentation was found to be less reliable in establishing the diagnosis in our patient cohort, as the presence of this symptom was only present in 40%. The presence of elevated WCC and markers of inflammation are sensitive markers of the condition, especially in those with atypical presentations. In our patient cohort, raised ESR and/or CRP were seen in over 95% of cases, with no significant differences seen between native and PJIs (p= 0.524). Despite this, acute phase reactants are known to unreliably discriminate between bacterial and non-bacterial sources for inflammation and have thus been described as being more helpful and practical for monitoring purposes.^3^

The knee is known to be the most commonly affected joint according to various studies; ^12,16^ this was reflected well in our study with over 60% of both native and PJIs occurring in knees (*Table 1*). 50 cases of prosthetic hip infections were encountered in the 10-year period of observation; this was in contrast to only 7 cases of native hip infection encountered which was shown to be statistically significant (p= <0.001). Whilst the hip has been described in several studies to be a common site for native joint sepsis, this was not the case in our patient cohort.^17^

Several predisposing factors for the development of joint sepsis are now known, including advanced age, diabetes mellitus, recent joint surgery, previous joint disease (such as osteoarthritis and RA), skin infection, intravenous drug use, alcoholism and prior intra-articular glucocorticoid injection.^15^ Each factor appears to have only a modest independent impact, with the presence of several concomitant risk factors appearing to substantially increase the risk of joint sepsis. Diabetes mellitus is a well-known risk factor for joint sepsis and was commonly encountered in both native and prosthetic infections (22.6% and 24.6% respectively) in this cohort, with no significant differences between the two (p= 0.771). Bacteria have also been shown to localize more in joints with pre-existing arthritis, especially in the presence of synovitis. It is interesting to note the very low incidence of septic arthritis in patients with chronic inflammatory arthritis such as RA in our cohort; although more cases of PJI were seen in RA patients (n=8) compared to native joint sepsis (n=2), this was not shown to be statistically significant (p= 0.108). The association of septic arthritis with inflammatory arthritis has long been recognized, but published data in this regard is still lacking to this day. The increased risk has been postulated to be a result of on-going inflamed synovium as well the effects of disease modifying antirheumatic drugs on the immune system.^18^ One UK based study using data from the British Society for Rheumatology Biologics Register reported that prior joint surgery was a risk factor in RA patients and that those treated with anti-tumour necrosis factor therapy had an incidence rate of 4.2 per 1000 patient years.^19^ The reasons for the low incidence rates of septic arthritis in the Maltese RA population remains unclear but could possibly be a result of improved treatment strategies and reduced rates of active inflammatory arthritis. Unfortunately, no formal epidemiological studies on RA have been carried out in Malta to date. Similarly, low rates of septic arthritis in patients with a history of illicit intravenous drug use were encountered in our patient cohort, accounting for only 5.6% of native joint sepsis. This contrasts to the findings in one recently published nationwide study conducted in the US which reported that the proportion of patients with intravenous drug use-related joint sepsis increased to 11% in 2013.^20^ Reasons for this disparity could include differences in rates of illicit drug users between Malta and the US, as well as the exclusion in our study of cases of osteomyelitis without definite joint involvement.

It is well known that *Staphylococcus aureus* is the commonest bacterium to infect adult joints in all age and risk groups, followed by other gram-positive organisms such as streptococci.^1,21–23^ This was reflected well in our study with *Staphylococcus aureus* being the most frequently cultured bacterium in both native (61.4%) and PJIs (56.2%), followed by streptococci spp. (17%) as the second commonest cause for native joint sepsis. The presence of MRSA is becoming an emergent problem and is becoming increasingly more common in cases of community-onset adult septic arthritis in Western populations.^22,23^ Although no appreciable change in incidence rates for prosthetic joint aspirates infected with MRSA were found throughout the entire 10 year period of observation, 10 of the 22 native joint aspirates with positive MRSA growths (45.5%) were cultured between 2016–2018. This coupled with the fact that 29.8% of patients in this cohort were hospitalised during the 3 months prior to the onset of joint sepsis (as opposed to 65.2% of cases of PJI) suggests an increasing trend of methicillin resistance in the Maltese community. A study carried out in 2013 found a very high prevalence of MRSA carriage in the Maltese community, compared to other EU countries, with positive strains encountered in up to 10% of hospital admissions.^24^

Similar infection rates for all organisms cultured was observed when native joint infections were compared to PJIs, apart from the much higher rates of clinically significant prosthetic infections with CoNS (23.1% vs 4.5% for PJIs and native joint infections respectively, p= <0.001) – this is also in-keeping with other studies carried out in Europe and the US which showed that CoNS together with *Staphylococcus aureus* are the most common isolated organisms in PJIs, accounting for up to two-thirds of cases.^25,26^ Higher rates of infection with gram negative organisms was also noted in prosthetic joints (23.1%) compared to native joints (15.9%), but this was not shown to be statistically significant (p= 0.224). It is also interesting to note that no cases of gonococcal arthritis were encountered in this study. Gonorrhoea has become rare in Western countries, with 2016 data from the European Centre for Disease Prevention and Control giving rates of 18.8 cases per 100,000 population in Europe, up to 3% of which may lead to systemic complications such as gonococcal arthritis.^27^ Prevalence rates are highest in younger patients, with over 70% of cases seen in the 15–34 year age group. Disseminated gonococcal infection presents with skin lesions in up to 60% of cases and is often polyarticular in nature. The reason behind the absence of gonococcal arthritis in the Maltese community is uncertain but is thought to be a reflection of the low local incidence of the disease together with the difficulties encountered when culturing the organism, as *Neisseria gonorrhoea* is very sensitive to environmental conditions.^27^ We believe the absence of confirmed cases is reflected by a patient cohort that is predominantly over 40 years in age, the relative absence of identified polyarticular cases and the low number of patients requiring treatment with cephalosporins. It remains possible however that some cases were overlooked as they might have presented with other symptoms and were treated promptly.

There is general consensus that the mainstay of management is adequate drainage and treatment with antimicrobials. The choice of empirical antibiotics remains debatable as there have been no randomised controlled trials that compare antimicrobial regimens to date, with little evidence to guide optimal antibiotic duration.^28^ The empirical agent of choice is dependent on clinical presentation and the presence of clinical risk factors such as immunocompromised states or history of intravenous drug abuse. Medical treatment is ultimately pathogen directed and guided by the results of gram stain and antimicrobial susceptibility testing on culture results. Locally, empirical antibiotic selection is usually based on local guidelines and discussion with hospital infectious diseases specialists, after assessing risk for gram negative sepsis (elderly, frail, recurrent urinary tract infections and recent abdominal surgery) and MRSA status (known MRSA, recent inpatient, nursing home resident, leg ulcers or catheters) along with treatment tolerability and allergies. Given the high percentages for MRSA strains in both cohorts, as well as the community, it would be prudent to cover locally with antimicrobials that have activity against MRSA until bacteriological identification by means of culture. Over 80% were started on a combination of at least two types of antibiotics.

Synovial drainage strategies in native joint sepsis are based on retrospective studies as there are no randomized controlled studies evaluating joint drainage procedures making it difficult to establish evidence-based recommendations for surgical management.^28^ Limited data from small retrospective studies suggests that surgical management is not superior to medical management with closed-needle aspiration in terms of both morbidity and mortality. ^29,30^ Over 75% of native joint infections in this cohort had one or more joint drainage procedures via needle arthrocentesis and/or surgical drainage.

In the case of PJIs, management normally consists of joint surgery and prolonged antimicrobial therapy with agents that suppress or treat biofilm bacteria. The approach depends on the age of the prosthesis, condition of the implant, patient characteristics and is ultimately guided by antimicrobial susceptibility testing on culture results. Recommendations are that well-fixed prostheses less than 30 days old or within 3 weeks of symptom onset, and that are susceptible to oral antibiotics in the absence of signs of a sinus tract undergo DAIR.^31^ In those patients where a DAIR is not feasible, a one- or two-stage exchange surgery may be offered. Resection arthroplasty with or without arthrodesis has limited indications in select patients. Non-surgical management is not recommended and should be considered only for those who are medically unfit or where patient preferences preclude surgery.^31, 32^ Over 50% of patients reviewed had undergone DAIR suggesting that over half of cases were either acute in nature or occurred in the acute post-operative period. This is also evident by the fact that 65.2% of PJIs were recently hospitalized prior to developing joint sepsis.

Mortality due to septic arthritis is usually dependant on the organism involved as well as the presence of advanced age and clinical comorbidities. Mortality rates in most series vary from 5–15%.^4,33^ *Staphylococcus aureus* is the usual culprit with higher rates reported in cases of methicillin resistance.^33^ The mortality rate for both native and PJIs in our cohort was found to be similar over the 10-year period of observation, around 5.7% (p=1.000). All deaths that were attributed directly to joint sepsis in this cohort involved elderly patients that suffered from one or more clinical comorbidity. 11 out of 15 deaths occurred in patients with a history of diabetes mellitus. Death due to Staphylococcus aureus occurred in 12 cases (80%).

There were several limitations that may have affected this study. Firstly, the rarity of septic arthritis meant that a relatively small number of reported cases were seen over the 10-year period of observation. Secondly, the retrospective nature for case evaluation may have resulted in higher degrees of inaccuracies during the data collection and interpretation process due to potential errors in recordkeeping. Thirdly, this was a hospital-based study and is thus not truly reflective of the Maltese population in its entirety – despite Mater Dei Hospital being the only hospital with dedicated microbiology and infectious disease clinicians in the Maltese Islands, it remains possible that few cases may have potentially been treated in private hospitals or within the community without microbiological advice; Similarly, this study does not take into account potentially fatal cases of septic arthritis treated out of hospital.

## CONCLUSIONS

Septic arthritis is a rare but important disease with a mortality rate of around 5.7% in our patient cohort. *Staphylococcus aureus* was the most commonly encountered causative agent in line with epidemiological findings in many regions of the Western world. A substantial proportion of cases, including those from the community, were methicillin resistant, implying that empirical antibiotic should provide cover for potential MRSA until the offending bacterium is isolated. Absence of fever and normal white cell count do not exclude the diagnosis so clinical awareness remains pivotal in reaching the correct diagnosis and timely management. More than half of patients had a predisposing risk for joint sepsis, implying that clinicians should be more vigilant in patients presenting with an acute swollen joint in the presence of risk factors. Elderly patients with clinical comorbidities have the highest mortality risk. The adage that a single hot swollen joint is septic until proven otherwise is a useful recommendation to ensure that a high index of suspicion is maintained.

## ABBREVIATIONS

CHF: Congestive heart failure
CKD: Chronic kidney disease
CoNS: Coagulase negative Staphylococci
CRP: C-Reactive protein
DAIR: Debridement antibiotics and implant retention
DM: Diabetes mellitus
ESR: Erythrocyte sedimentation rate
ICD-10: International Classification of Diseases 10^th^ Revision
IHD: Ischaemic heart disease
IQR: Interquartile range
MRSA: Methicillin resistant Staphylococcus aureus
MSSA: Methicillin sensitive Staphylococcus aureus
PJI: Prosthetic joint infection
RA: Rheumatoid arthritis
SE: Standard error
WCC: White cell count
